# Clinical Outcomes After Radioiodine Therapy, According to the Method of Preparation by Recombinant TSH vs. Endogenous Hypothyroidism, in Thyroid Cancer Patients at Intermediate-High Risk of Recurrence

**DOI:** 10.3389/fnume.2021.785768

**Published:** 2021-11-30

**Authors:** Cynara Rena Salmont Higuchi, Paula Fernanda, Paulo Alonso Jurnior, Fernanda Accioly Andrade, Rossana Corbo, Mario Vaisman, Fernanda Vaisman, Daniel Bulzico

**Affiliations:** ^1^Endocrinology Service, Instituto Nacional do Cancer do Rio de Janeiro, Rio de Janeiro, Brazil; ^2^Endocrinology Service, Faculdade de Medicina, Universidade Federal do Rio de Janeiro, Rio de Janeiro, Brazil

**Keywords:** thyroid cancer, recombinant human TSH, radioiodine, intermediate to high risk, ablation

## Abstract

**Background:** To effectively treat differentiated thyroid carcinoma (DTC) with radioiodine therapy (RAI), it is necessary to raise serum thyrotropin levels, either by thyroid hormone withdrawal (THW) or by administration of recombinant human TSH (rhTSH). The use of rh-TSH is controversial in DTC patients at intermediate to high risk of recurrence. Even more controversial is the question of whether is alters progression-free survival rates and overall survival in more aggressive patients.

**Objective:** The primary objective of this study was comparing clinical outcomes according to the method of preparation of RAI in intermediate to high DTC patients who presented progression of structural disease.

**Methods:** This retrospective study included 81 patients with initial intermediate to high DTC and progression of structural disease at the end of follow-up. In 21 patients, all RAI treatments were done with only rhTSH stimulation. In 11, RAI treatments were done either with thyroid hormone withdrawal (THW) or rhTSH. In 49 patients, all RAI treatments were done only THW.

**Results:** After a median follow-up time of 83 months, there were no statistical differences in the clinical outcomes (status of structural disease at the end of the follow-up, death rate, overall survival curve, and progression-free survival curve).

**Conclusions:** Preparation for RAI therapy using either rhTSH stimulation or THW was associated with no inferiority in the clinical outcomes in progressive DTC patients at higher risk of recurrence.

## Introduction

In general, differentiated thyroid carcinoma (DTC) presents an indolent course and excellent prognosis, with a 10-year survival rate that exceeds 90–95%. This overall survival rate decreases when distant metastasis is present. As described in the AJCC 8th edition, the mortality rate is 50% in stage IV patients over the first 5 years of follow-up (1, 2).

The management of patients with DTC at intermediate to high risk of recurrence consists of total thyroidectomy with or without lymph node dissection, followed by radioiodine therapy (RAI). RAI adjuvant therapy is routinely recommended after total thyroidectomy for intermediate to high DTC patients because it is associated with increased overall survival and disease-free survival (2).

Two methods of preparation for RAI can be used to achieve the goal TSH of >30 mIU/L. One method is endogenous hypothyroidism after thyroid hormone withdrawal (THW) for a period of 3–4 weeks, the other method is exogenous stimulation with the administration of recombinant human TSH (rhTSH). The use of rhTSH is related to a better quality of life in the short term because it avoids the symptoms of hypothyroidism resulting from THW (2, 3).

In intermediate to high DTC patients, rhTSH preparation for RAI adjuvant treatment is not routinely recommended, especially if distant metastases are known or suspected. According to the current American Thyroid Association (ATA) guidelines, more data from studies with long-term outcomes are needed before preparation with rhTSH can be recommended in this group of patients (2). However, this preparation can be considered in intermediate to high DTC patients with significant comorbidities, such as severe lung disease, congestive heart failure, coronary artery disease, psychiatric disorders, and others. In these situations, endogenous hypothyroidism resulting from THW may be poorly tolerated and lead to serious adverse events (2, 4). The use of rhTSH can also be considered in patients who do not have an increased TSH after TWH, such as in central hypothyroidism and those with a high tumor burden (2, 5).

In recent years, there has been an increase in retrospective studies suggesting that rhTSH-stimulated RAI therapy may be effective in treating ATA high-risk DTC patients and/or RAI avid distant metastasis (3, 4, 6–9), as well as in remnant ablation, in patients with primary T4 tumor (10). However, it is still questionable whether the radiation activity delivered to DTC metastases after rhTSH is lower compared to preparation by THW (11).

Furthermore, no randomized prospective trials have been performed evaluating the RAI preparation in these patients, and there are no data in the literature comparing the method of RAI preparation (rhTSH vs. endogenous hypothyroidism) in ATA high-risk DTC patients with structural disease progression. It is known that around 30–40% of patients with the structural disease may progress during follow-up (2), however, it is not yet studied if the preparation for RAI could impact this outcome.

The preparation of RAI with rhTSH has been performed at the Instituto Nacional de Cancer (INCA) in ATA high-risk DTC patients since 2010. In this institution, the preparation of RAI with rhTSH is not indicated only in patients with known distant metastasis to the spine, skull, central nervous system (CNS), in which the abrupt increase of TSH could cause spinal cord compression or damage to the CNS. In these patients, the RAI preparation is always performed by endogenous hypothyroidism, through THW.

Thus, the aim of this study is to retrospectively compare the clinical outcomes, according to the method of preparation of the RAI, in ATA intermediate to high-risk DTC patients, who presented progression of structural disease.

## Subjects and Methods

### Subjects

The study was carried out at the Oncological Endocrinology Sector of INCA, through a retrospective analysis of medical records of patients with DTC at high risk of recurrence, with the progression of structural disease after RAI. To identify the patients, an active search was carried out in the historical records of the Nuclear Medicine and Endocrinology services of INCA, including patients who underwent RAI between 08/01/1997 and 06/05/2019. This study was approved by the local Research Ethics Committees.

A total of 81 patients were included in the study. Inclusion criteria were: age over 18 years, histologically confirmed diagnosis of DTC after total thyroidectomy or near-total thyroidectomy, clinical staging of ATA high risk of recurrence; at least one RAI activity at the INCA, minimum follow-up of 12 months and presence of progression of structural disease after RAI anytime during follow-up.

Regarding the method of RAI preparation, patients were divided into three groups. Group 1 was composed of patients who performed RAI with rhTSH only. Group 2 was composed of patients who performed more than 1 RAI, and whose preparation was sometimes with rhTSH and sometimes with THW. Group 3 was composed of patients who performed RAI and whose preparation was only endogenous hypothyroidism after THW for 3–4 weeks. All patients treated with RAI at our center were instructed in a low-iodine diet.

### Variables Studied

As for the clinical characteristics of the patients, the variables studied were age at diagnosis, gender, the histological subtype of DTC, tumor size, and TNM staging, according to American Joint Committee on Cancer (AJCC) 8th edition (2018). The diagnosis of distant metastasis (DM) was classified as distant metastasis at the time of initial disease staging, distant metastasis identified during longitudinal follow-up, or no distant metastasis. The distribution of distant metastases (lung and/or bone) was also evaluated.

Regarding the presentation of distant metastasis, it was considered as initial distant metastasis if the presence of metastasis was found in imaging or histopathological exam before or at the time of diagnosis of DTC; evidence of RAI avid distant metastasis (DM) found on posttherapy whole-body scanning (RxWBS); or high stimulated thyroglobulin (Tg) observed in the first RAI, suggestive of distant metastasis and confirmed in the first imaging exam performed after RAI. Follow-up metastasis was considered when the patient had a normal imaging exam after the first RAI and the subsequent appearance of a metastatic lesion. The absence of distant metastasis was considered in those patients with no diagnosis of distant metastasis by functional examination (diagnostic RAI whole-body scanning, PET/CT, bone scintigraphy) or anatomical imaging (CT or MRI).

As for treatment, the following variables were evaluated: initial activity of the first RAI, posttherapy whole-body scanning (RxWBS) after the first RAI, RAI preparation, cumulative radioiodine activity, additional therapies. Additional therapies were those performed during follow-up, such as surgical intervention for patients with persistent/recurrent loco-regional disease, external radiotherapy, multikinase inhibitors (MKI), metastasectomy, and bisphosphonates (zolendronic acid).

And finally, variables were evaluated regarding clinical outcomes: dynamic risk stratification at the end of follow-up, according to the ATA guideline 2015; structural disease status at the end of follow-up, through the evaluation of the last Tg and antithyroglobulin and available imaging tests; progression-free survival; overall survival (at death); follow-up time; and death rate.

Due to the profile of the patients included in the study, none of them had an absence of disease at the end of follow-up. Regarding the status of the structural disease at the end of follow-up, stable disease was considered when there was no structural progression in the last year of follow-up, and disease progression when there was an increase or appearance of a new structural lesion in the last year of follow-up, as well as the need for additional therapy during this period or death.

Two different analyses were performed: Comparing the three proposed groups: THW only, rhTSH only, and a mixed group; and a second analysis comparing only patients prepared exclusively with one modality.

### Statistical Methods

Median, with minimum and maximum, was used to express continuous variables. Percentages and frequencies were used for categorical variables. Categorical comparisons were analyzed using the chi-square test and continuous variables were compared using the median test and Kruskal–Wallis test. Kaplan Meier and log-rank were used for the overall survival and progression-free survival analyses. *P* < 0.05 were considered statistically significant. Analyses were performed using SPSS for Windows software (version 20.0; SPSS, Inc., Chicago, IL).

## Results

### Clinicopathological Characteristics and Clinical Outcomes of the Entire Cohort

Eighty-one patients were included in the study. Clinical characteristics at the time of diagnosis and clinical outcomes at the end of follow-up are described in Tables 1, 2.

**Table 1:** Clinical characteristics of the entire cohort.

	***n* = 81**
Age (year)	
Median (range)	63 (24–83)
Gender [*n* (%)]	
Female	50 (61.7%)
Male	31 (38.3%)
Histology [*n* (%)]	
PTC	36 (44.5%)
FTC	18 (22.2%)
PTCFV	10 (12.3%)
PTC, other variants	5 (6.2%)
Hürthle cell Ca	6 (7.4%)
Poorly differentiated carcinoma	6 (7.4%)
Size (cm)	
Median (range)	4.7 (0.4–12)
*T* [*n* (%)]	
T1	13 (16%)
T2	13 (16%)
T3	19 (23.5%)
T4	24 (29.7%)
Tx	12 (14.8%)
*N* [*n* (%)]	
0	4 (4.9%)
N1a	12 (14.8%)
N1b	21 (25.9%)
Nx	44 (54.4%)
Discovery of distant **metastasis [*****n*** **(%)]**	
At initial presentation	66 (81.5%)
During follow up	8 (9.9%)
No distant metastasis	7 (8.6%)
Distribution of distant metastasis [*n* (%)]	
M0/Mx	15 (18.5%)
Lung	45 (55.6%)
Bone	10 (12.3%)
Lung and bone	11 (13.6%)
Activity of the first RAI (mCi)	
Median (range)	200 (100–300)
RxWBS after first RAI [*n* (%)]	
Anterior cervical	34 (42%)
Lateral cervical	16 (19.8%)
Distant disease	29 (35.7%)
Unknown	2 (2.5%)
Preparation for RAI treatment(s) [*n* (%)]	
Group 1	21 (25.9%)
Group 2	11 (13.6%)
Group 3	49 (60.5%)
RAI cumulative administered activity (mCi)	
Median (range)	250 (100–850)
Additional therapies [*n* (%)]	69 (85.2%)
PET [*n* (%)]	
No FDG uptake	6 (7.4%)
FDG uptake	68 (84%)
Unrealized	7 (8.6%)

*PTC, papillary thyroid carcinoma; FTC, follicular thyroid carcinoma; PTCFV, papillary thyroid carcinoma, follicular variant; PTC, other variants: papillary thyroid carcinoma, other variants; Hürthle cell Ca, Hürthle cell carcinoma; poorly differentiated Ca, poorly differentiated carcinoma. T1, tumor ≤ 2.0 cm; T2, tumor > 2 and ≤ 4.0; T3, tumor > 4.0 cm restricted to thyroid or tumor of any size with extra-thyroid extension involving only musculature; T4, tumor of any size with gross extra-thyroid extension; Tx, absence of tumor size data; N1a, lymph node metastasis to central compartment; N1b, lymph node metastasis to lateral compartment; Nx, unassessed lymph node metastasis; M0/Mx, absence of distant metastasis or unassessed distant metastasis. RAI, radioiodine therapy; RxWBS, posttherapy whole-body scanning; RAI 1, first radioiodine therapy; PET, positron emission tomography; Group 1, RAI preparation after recombinant TSH (only); Group 2, RAI performed after recombinant TSH or thyroid hormone withdrawal at different times; Group 3, RAI preparation after thyroid hormone withdrawal (only)*.

**Table 2:** Clinical outcomes of the entire cohort.

	***n* = 81**
Follow up (mo)	
Median (range)	83 (13–281)
Dynamic risk stratification [*n* (%)]	
Structural incomplete response	80 (98.8%)
Indeterminate response	1 (1.2%)
Structural disease status [*n* (%)]	
Stable disease	11 (13.6%)
Progressive structural disease	70 (86.4%)
Death [*n* (%)]	
No	39 (48.1%)
Yes	42 (51.9%)

The median age was 63 years (range 24–83) and 61.7% were women. The most frequent histological subtype was papillary thyroid carcinoma (PTC), present in 36 patients (44.5%); followed by follicular thyroid carcinoma (CFT) in 18 patients (22.2%); papillary thyroid carcinoma, follicular variant (PTCFV) in 10 patients (12.3%); Hürthle cell carcinoma in six patients (7.4%); poorly differentiated carcinoma in six patients (7.4%); and PTC, other variants in five patients (6.2%).

The median tumor size was 4.7 cm (range 0.4–12), and the initial staging T4 was the most frequent, present in 24 patients (29.7%). In total 21 patients (25.9%) have been classified N1b lymph node metastasis and 12 patients (14.8%) N1a.

Regarding the diagnosis of distant metastasis, 81.5% were diagnosed metastasis at initial presentation, 9.9% distant metastasis during follow-up, 8.6% no distant metastasis, and were considered to have locally advanced cancer. The most common site of metastasis at initial presentation was lung, present in 45 patients (55.6%), followed by lung and bone in 11 (13.6%), and bone in 10 (12.3%).

The median activity of the first RAI was 200 mCi (range 100–300) and 29 patients (35.7%) had RAI avid distant metastasis (DM) found on posttherapy whole-body scanning (RxWBS). Regarding RAI preparation, 21 patients (25.9%) were in group 1, 11 patients (13.6%) were in group 2, and 49 patients (60.5%) were in group 3.

The median follow-up time was 83 months (range 13–281); 80 patients (98.8%) had an incomplete structural response at the end of follow-up and 1 patient had an indeterminate response. At the last evaluation, 70 patients (86.4%) had progressive structural disease or death and 11 patients (13.6%) had stable disease. Of the 81 patients, 42 (51.9%) died and 39 (48.1%) did not.

### Clinicopathological Features, According to RAI Preparation

As described above, 21 patients (25.9%) were prepared for RAI only with rhTSH (group 1), 11 patients (13.6%) performed more than one RAI and were prepared sometimes with THW and sometimes with rh-TSH (group 2), and 49 patients (60.5%) performed RAI only after THW (group 3). Of the patients in group 2, 10 performed the first RAI after THW, and the others RAIs after rhTSH. Only one patient in group 2 had the first RAI after rhTSH, and the others RAIs after THW.

The clinical characteristics based on the method of preparation for RAI treatment are given in Table 3. When evaluating the clinical characteristics of the three groups according to age, sex, histology, tumor size, TNM staging, diagnosis and distribution of distant metastasis, activity, and RxWBS of the first RAI, there was no statistical difference according to the method of preparation for RAI.

**Table 3:** Clinicopathological features, according to RAI preparation three groups.

	**Group 1**	**Group 2**	**Group 3**	***p*-value**
	***n* = 21**	***n* = 11**	***n* = 49**	
Age (year)				
Median (range)	63 (24–83)	57 (46–80)	63 (31–79)	0.85
Gender [*n* (%)]				
Female	12 (57.1%)	9 (81.8%)	29 (59.2%)	0.33
Male	9 (42.9%)	2 (18.2%)	20 (40.8%)	
Histology [*n* (%)]				
PTC	9 (42.8%)	5 (45.3%)	22 (44.8%)	0.57
FTC	5 (23.8%)	4 (36.4%)	9 (18.4%)	
PTCFV	1 (4.8%)	2 (18.2%)	7 (14.3%)	
PTC, others variants	3 (14.3%)	0	2 (4.1%)	
Hürthle cell Ca	1 (4.8%)	0	5 (10.2%)	
Poorly differentiated carcinoma	2 (9.5%)	0	4 (8.2%)	
Size (cm)				
Median (range)	4.4 (1–12)	3.2 (0.4–9)	4.5 (1.1–11)	0.65
*T* [*n* (%)]				
T1	5 (23.8%)	2 (18.2%)	6 (12.2%)	0.26
T2	4 (19%)	0	9 (18.4%)	
T3	3 (14.3%)	2 (18.2%)	14 (28.6%)	
T4	8 (38.1%)	2 (18.2%)	14 (28.6%)	
Tx	1 (4.8%)	5 (45.4%)	6 (12.2%)	
*N* [*n* (%)]				
0	1 (4.8%)	0	3 (6.1%)	0.12
N1a	3 (14.3%)	1 (9.1%)	8 (16.3%)	
N1b	9 (42.8%)	0	12(24.5%)	
Nx	8 (38.1%)	10 (90.9%)	26 (53.1%)	
Discovery of distant metastasis [*n* (%)]				
At initial presentation	19 (90.5%)	10 (90.9%)	37 (75.5%)	0.27
During follow up	2 (9.5%)	1 (9.1%)	5 (10.2%)	
No distant metastasis	0	0	7 (14.3%)	
Distribution of distant metastasis [*n* (%)]				
M0/Mx	2 (9.5%)	1 (9.1%)	12 (24.5%)	0.49
Lung	15 (71.4%)	6 (54.5%)	24 (49%)	
Bone	1 (4.8%)	2 (18.2%)	7 (14.3%)	
Lung and bone	3 (14.3%)	2 (18.2%)	6 (12.2%)	
Activity of the first RAI (mCi)				
Median (range)	150 (150–300)	175 (100–250)	200 (100–300)	0.66
RxWBS of the first RAI [*n* (%)]				
No uptake	0	0	1 (2%)	0.52
Anterior cervical	7 (33.3%)	3 (27.3%)	24 (49%)	
Lateral cervical	5 (23.8%)	1 (9.1%)	10 (20.4%)	
Distant disease	9 (42.9%)	6 (54.5%)	14 (28.6%)	
Unknown	0	1 (9.1%)	0	

*PTC, papillary thyroid carcinoma; FTC, follicular thyroid carcinoma; PTCFV, papillary thyroid carcinoma, follicular variant; PTC, other variants: papillary thyroid carcinoma, other variants; Hürthle cell Ca, Hürthle cell carcinoma; poorly differentiated Ca, poorly differentiated carcinoma. T1, tumor ≤ 2.0 cm; T2, tumor > 2 and ≤ 4.0; T3, tumor > 4.0 cm restricted to thyroid or tumor of any size with extra-thyroid extension involving only musculature; T4, tumor of any size with gross extra-thyroid extension; Tx, absence of tumor size data; N1a, lymph node metastasis to central compartment; N1b, lymph node metastasis to lateral compartment; Nx, unassessed lymph node metastasis; M0/Mx, absence of distant metastasis or unassessed distant metastasis. RAI, radioiodine therapy; RxWBS, posttherapy whole-body scanning; PET, positron emission tomography; Group 1, RAI preparation after recombinant TSH (only); Group 2, RAI performed after recombinant TSH or thyroid hormone withdrawal at different times; Group 3, RAT preparation after thyroid hormone withdrawal (only)*.

The frequency of distant metastasis at initial presentation was 90.5% in group 1, 90.9% in group 2, and 75.5% in group 3. The most common site of metastasis at initial presentation in all 3 groups was the lung, present in 15 patients in group 1 (71.4%), 6 patients (54.5%) in group 2, and 24 patients (49%) in group 3.

The median activity of the first RAI was 150 mCi, 175 mCi, and 200 mCi in groups 1, 2, and 3, respectively. Regarding RAI cumulative administered activity, there was a statistical difference between the 3 groups (*p* 0.003), with the median in group 1 being 200 mCi (range 150–850), group 2 400 mCi (range 200–850), and group 3, 250 mCi (range 100–850). In group 2, all patients performed more than one RAI (100%), while only 23.8 and 42.8% performed more than one RAI in groups 1 and 3, respectively. When performing the paired comparison of the median accumulated radioiodine activity, there was no statistical difference between groups 1 and 3 (*p* 0.389) and 2 and 3 (*p* 0.062), but there was a statistical difference between groups 1 and 2 (*p* 0.002).

Additional therapies were performed in 17 patients in group 1 (81%), 10 patients in group 2 (90.9%), and 42 patients in group 3 (85.7%), and there was no statistical difference between the three groups (*p* 0.74).

Comparing THW only with rhTSH only, also no differences among the groups were seen (Table 4).

**Table 4:** Clinicopathological features and according to RAI preparation.

	**rhTSH**	**THW**	***p*-value**
	***n* = 21**	***n* = 49**	
Age (year)			
Median (range)	63 (24–83)	63 (31–79)	0.79
Gender [*n* (%)]			
Female	12 (57.1%)	29 (59.2%)	0.87
Male	9 (42.9%)	20 (40.8%)	
Histology [*n* (%)]			
PTC	9 (42.8%)	22 (44.8%)	0.54
FTC	5 (23.8%)	9 (18.4%)	
FVPTC	1 (4.8%)	7 (14.3%)	
PTC, others variants	3 (14.3%)	2 (4.1%)	
Hürthle cell Carcinoma	1 (4.8%)	5 (10.2%)	
Poorly differentiated carcinoma	2 (9.5%)	4 (8.2%)	
Size (cm)			
Median (range)	4.4 (1–12)	4.5 (1.1–11)	0.99
*T* [*n* (%)]			
T1	5 (23.8%)	6 (12.2%)	0.72
T2	4 (19%)	9 (18.4%)	
T3	3 (14.3%)	14 (28.6%)	
T4	8 (38.1%)	14 (28.6%)	
Tx	1 (4.8%)	6 (12.2%)	
*N* [*n* (%)]			
0	1 (4.8%)	3 (6.1%)	0.49
N1a	3 (14.3%)	8 (16.3%)	
N1b	9 (42.8%)	12(24.5%)	
Nx	8 (38.1%)	26 (53.1%)	
Discovery of distant metastasis [*n* (%)]			
At initial presentation	19 (90.5%)	37 (75.5%)	0.18
During follow up	2 (9.5%)	5 (10.2%)	
No distant metastasis	0	7 (14.3%)	
Distribution of distant metastasis [*n* (%)]			
M0/Mx	2 (9.5%)	12 (24.5%)	0.24
Lung	15 (71.4%)	24 (49%)	
Bone	1 (4.8%)	7 (14.3%)	
Lung and bone	3 (14.3%)	6 (12.2%)	
Activity of the first RAI (mCi)			
Median (range)	150 (150–300)	200 (100–300)	0.79
RxWBS of the first RAI [*n* (%)]			
No uptake	0	1 (2%)	0.52
Anterior cervical	7 (33.3%)	24 (49%)	
Lateral cervical	5 (23.8%)	10 (20.4%)	
Distant disease	9 (42.9%)	14 (28.6%)	
Unknown	0	0	
RAI cumulative administered activity (mCi)			
Median (range)	200 (150–850)	250 (100–850)	0.21
Additional therapies [*n* (%)]	17 (81%)	42 (85.7%)	0.62
PET [*n* (%)]			
No FDG uptake	1 (4.8%)	2 (4.1%)	0.72
FDG uptake	17 (80.9%)	43 (87.7%)	
Unrealized	3 (14.3%)	4 (8.2%)	

*PTC, papillary thyroid cancer; FTC, follicular thyroid cancer; FVPTC, follicular variant papillary thyroid cancer; RAI, radioactive iodine; RxWBS, post-therapy whole body scan*.

### Clinical Outcomes Based on Method of Preparation

The clinical outcomes at the end of follow-up based on the method of preparation for RAI treatment are given in Tables 5, 6. There was no statistical difference in progression-free survival, dynamic risk stratification, structural disease status, and death between the 3 groups. All 100% of patients in group 1, 100% of group 2, and 98% of group 3 had an incomplete structural response (*p* 0.71). At the last evaluation, 85.7% of patients in group 1, 100% of group 2, and 83.7% of group 3 had progressing structural disease (*p* 0.36). The frequency of deaths was 47.6% of group 1, 36.4% of group 2, and 57.1% of group 3 (*p* 0.41).

**Table 5:** Clinical outcomes, according to the RAI preparation.

	**Group 1**	**Group 2**	**Group 3**	***p*-value**
	***n* = 21**	***n* = 11**	***n* = 49**	
Follow up (mo)				
Median (range)	61 (19–149)	125 (29–281)	88 (13–241)	0.014
Progression-free survival (mo)				
Median (range)	21 (7–68)	55 (23–225)	38 (2–121)	0.10
Dynamic risk stratification [*n* (%)]				
Structural incomplete response	21 (100%)	11 (100%)	48 (98%)	0.71
Indeterminate response	0	0	1 (2%)	
Structural disease status [*n* (%)]				
Stable disease	3 (14.3%)	0	8 (16.3%)	0.36
Progressive structural disease	18 (85.7%)	11 (100%)	41 (83.7%)	
Death [*n* (%)]				
No	11 (52.4%)	7 (63.6%)	21 (42.9%)	0.41
Yes	10 (47.6%)	4 (36.4%)	28 (57.1%)	

**Table 6:** Clinical outcomes, according to the RAI preparation.

	**rhTSH**	**THW**	***p*-value**
	***n* = 21**	***n* = 49**	
Follow up (mo)			
Median (range)	61 (19–149)	88 (13–241)	0.05
Progression-free survival (mo)			
Median (range)	21 (7–68)	38 (2–121)	0.37
Dynamic risk stratification [*n* (%)]			
Structural incomplete response	21 (100%)	48 (98%)	0.51
Indeterminate response	0	1 (2%)	
Structural disease status [*n* (%)]			
Stable disease	3 (14.3%)	8 (16.3%)	0.83
Progressive structural disease	18 (85.7%)	41 (83.7%)	
Death [*n* (%)]			
No	11 (52.4%)	21 (42.9%)	0.46
Yes	10 (47.6%)	28 (57.1%)	

There was a statistical difference (*p* 0.014) among the groups when evaluating the median follow-up time: 61 months (range 19–149), 125 months (range 29–281), and 88 months (range 13–241) in groups 1, 2, and 3, respectively. When performing the paired comparison between groups of the median follow-up time, there was no statistical difference between groups 1 and 3 (*p* 0.08), and 2 and 3 (*p* 0.28), but there was a statistical difference between groups 1 and 2 (*p* 0.002).

The median progression-free survival after RAI1 from groups 1, 2, and 3 was 21 (range 7–68), 55 (range 23–225), and 38 (range 2–121) months, respectively. There was no statistical difference in median progression-free survival after RAI1 between the groups (*p* 0.10).

**Figure 1:**
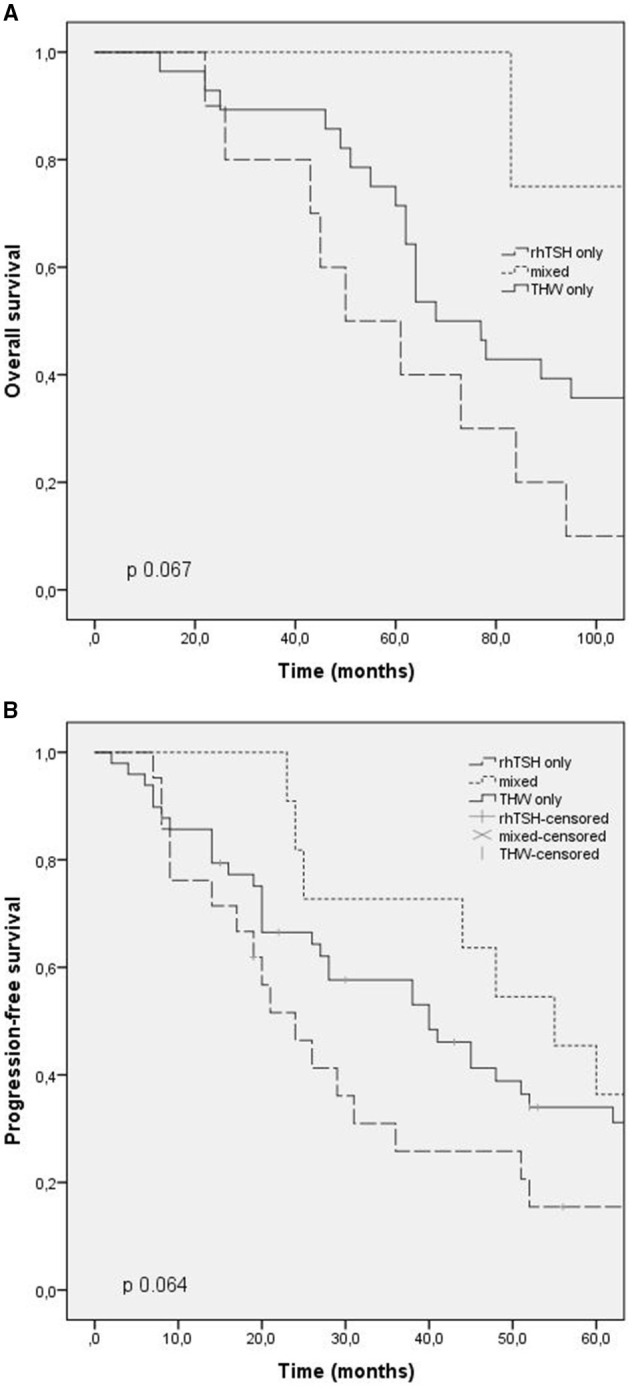
Outcomes according to RAI preparation (thyroid hormone withdrawal, TSH rh) and mixed. **(A)** Overall survival; **(B)** progression free survival.

**Figure 2:**
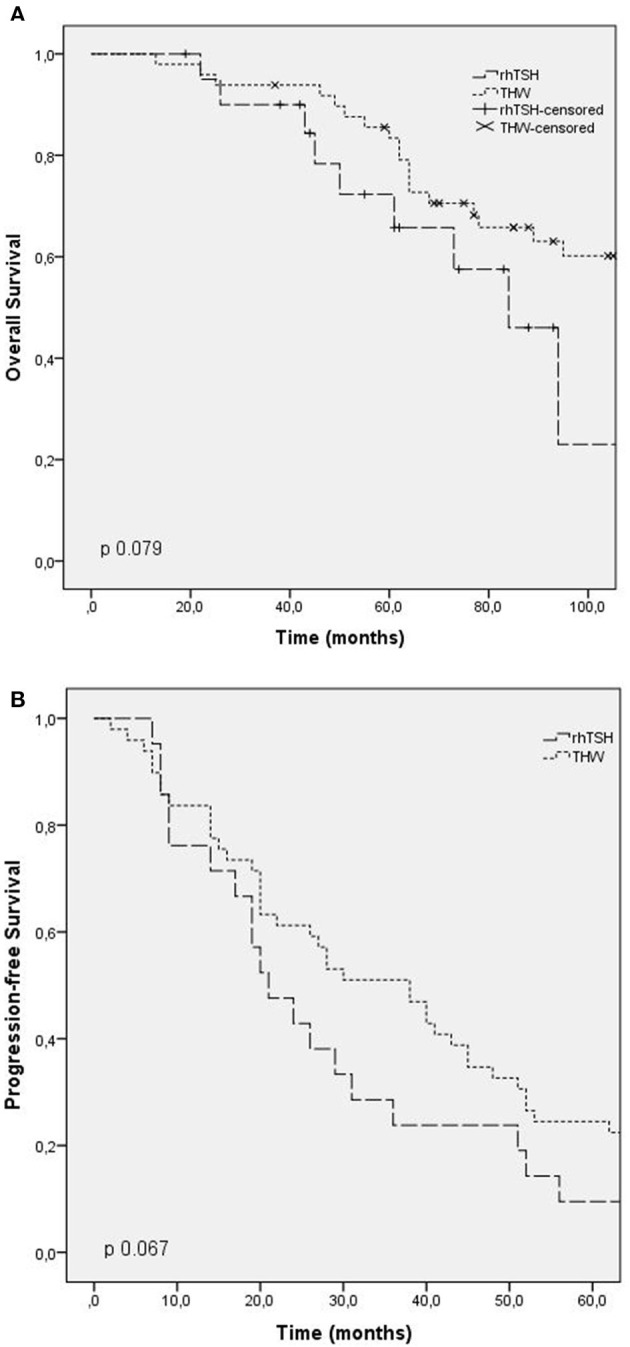
Outcomes according to RAI preparation (thyroid hormone withdrawal, TSH rh). **(A)** Overall survival; **(B)** progression free survival.

Overall survival time was evaluated in patients who died (42 patients). The median overall survival was 73 months (CI: 56.3–89.6), being 50 months in group 1 (CI: 25.2–74.7), 125 months in group 2 (CI: 31.9–218.1), and 68 months in group 3 (CI: 54.1–81.8). There was no statistical difference between the groups (*p* 0.06). The overall survival time curve in patients who died is shown in Figures 1A, 2A.

Progression-free survival after first RAI was evaluated in patients with progressing structural disease at the end of follow-up, and patients with stable disease were excluded. The median progression-free survival in these patients was 24 months (CI: 15.5–32.4) in group 1, 55 months in group 2 (CI: 37.7–72.2), and 40 months in group 3 (CI: 24.9–55). There was no statistical difference between the groups (*p* 0.6). The progression-free survival curve is shown in Figures 1B, 2B.

No adverse effect due to the use of TSHrh was reported nor major adverse effects due to THW.

Comparing groups 1 and 3, those with only one method of preparation, it was also possible to show no difference in outcomes (Table 6).

## Discussion

This study evaluating 81 patients with ATA high-risk DTC and structural disease progression shows non-inferiority in final clinical outcomes, according to the method of RAI preparation with rhTSH or endogenous hypothyroidism, in a population in which the use of rhTSH is still controversial. Even in these more aggressive patients that showed progression during follow-up, preparation for RAI did not interfere. Furthermore, using rhTSH with some caution, no adverse effect was reported.

In our study, there was a statistical difference in the accumulated radioiodine activity between the 3 groups, with group 2 showing the highest accumulated RAI activity, probably because all patients in this group performed more than one RAI. In the study by Tala et al. (6), which also showed no significant difference in outcomes attributed to the difference in preparation for RAI, the accumulated radioiodine activity was also higher in patients who underwent RAI after preparation with THW followed by rhTSH, when compared to the group who underwent preparation with THW only or rhTSH only (median 967 vs. 522 vs. 408 mCi, respectively, *p* 0.038). The author similarly justifies this fact due to the higher number of radioiodine treatments in the mixed group.

Regarding additional therapies, our study showed no statistical difference between the groups according to RAI preparation. Likewise, Klubo-Gwiezdzinska et al. (4) showed that the frequency of additional treatments, including other modalities applied, such as external beam radiation therapy, surgical excision of metastatic lesions, and treatment of patients with bone metastasis with zolendronic acid was similar between the groups that prepared with rhTSH vs. THW.

As described in the AJCC 8th edition (1), the mortality rate is 50% in stage IV patients over the first 5 years of follow-up. In our study, most patients were above 55 years old and already had distant metastasis at diagnosis. The death rate was high, similar to that described by the AJCC, and there was no statistical difference between the groups according to the RAI preparation. As expected by initial risk stratification, the persistence of structural disease at the end of follow-up also showed high rates, and most patients were classified as having an incomplete structural response and once again, these were not modified by different preparations for RAI.

In our study, the median progression-free survival was 21 months for the group prepared after rhTSH and 38 months in the group whose preparation was THW, and there was no statistical difference between the groups. In the study by Hugo et al. (4), the mean progression-free survival was 20 months in the group prepared after rhTSH and 24 months in the group after THW, and there was also no statistical difference between the groups. The progression-free survival in patients prepared after rhTSH in our study was similar to the study of Klubo-Gwiezdzinska et al. (6), although our cohort only presented ATA high-risk DTC patients with structural disease progression.

As with any retrospective study, there are some limitations that must be considered. First and foremost is the potential for a selection bias that may occur when prioritizing RAI after THW, instead of rhTSH, in patients with higher risk disease or metastasis to noble areas. However, this bias also occurs in other retrospective studies, and in our study, we observed no statistical difference in clinicopathological features between patients prepared with rhTSH and THW. Prospective, randomized studies would be necessary to address this potential bias.

In conclusion, in this retrospective study clinical outcomes were similar in the progressive high-risk DTC patients prepared for RAI after rhTSH only, or THW only, or different preparations. Thus, we did not observe inferiority in clinical outcomes according to RAI preparation in our cohort.

## Data Availability Statement

The raw data supporting the conclusions of this article will be made available by the authors, without undue reservation.

## Ethics Statement

The studies involving human participants were reviewed and approved by IRB Inca. Written informed consent for participation was not required for this study in accordance with the national legislation and the institutional requirements.

## Author Contributions

CH: data collection and writing. PF: data collection and data review. PJ and FA: data review and protocol design. RC and MV: data collection, manuscript review, and orientation. FV and DB: data collection, protocol design, manuscript review, and orientation. All authors contributed to the article and approved the submitted version.

## Conflict of Interest

The authors declare that the research was conducted in the absence of any commercial or financial relationships that could be construed as a potential conflict of interest.

## Publisher's Note

All claims expressed in this article are solely those of the authors and do not necessarily represent those of their affiliated organizations, or those of the publisher, the editors and the reviewers. Any product that may be evaluated in this article, or claim that may be made by its manufacturer, is not guaranteed or endorsed by the publisher.
